# P05.56. Prescribing nature: a content analysis of qualitative responses among park users by socio-demographics and patterns of use

**DOI:** 10.1186/1472-6882-12-S1-P416

**Published:** 2012-06-12

**Authors:** K Irvine, M Dallimer, S Warber

**Affiliations:** 1 De Montfort University, Leicester, United Kingdom; 2 University of Copenhagen, Copenhagen, Denmark; 3 University of Michigan, Ann Arbor, USA

## Purpose

Against a backdrop of increasing interest in ‘prescribing nature’ to promote health and well-being, this study investigated the extent to which adult users of urban greenspace endorsed health benefits and whether these effects differed between groups or frequency of use.

## Methods

*In situ* questionnaires were delivered face-to-face to 1108 users (54% response rate) of urban riverscapes in Sheffield, UK. First mentioned responses to an open-ended question about how they felt after leaving the area were content analysed. Resultant codes were grouped into themes and domains and quantified. Using Chi-square tests, response-patterns were compared across age, gender, visit frequency, number of people in the group, or whether walking a dog.

## Results

Comments from participants (92% European ethnicity, 62% male, aged 16-70+ years) primarily classified into emotional (38%), physical (34%), and spiritual (22%) domains of health. Cognitive (3%) and overall health (3%) domains were rarely first mentioned; mention of social health did not occur. Emotional included feelings within the self (happy, wonderful), amplified by feelings toward the greenspace itself (appreciation, sadness about condition). Relaxation featured prominently as a derived physical effect; tranquillity was strongly present within spiritual. Across the frequently mentioned domains, differences existed for gender (p=0.021), age (p<0.001) and whether participants were alone (p=0.001). Women more frequently mentioned spiritual; participants in their 20s or 50s identified aspects of physical health; being alone brought out feelings of place attachment. No significant differences were found for visit frequency (daily, weekly, monthly, less than monthly; p=0.304) or whether walking a dog (p=0.392).

## Conclusion

Greenspace provided multiple effects, primarily relaxation, positive emotions and tranquility, which promote human well-being. While specific groups of users obtained different effects, there was no evidence of a differentiated effect depending on visit frequency. This fine-grained analysis suggests useful guidelines for the development of nuanced, rather than generic, ‘nature prescriptions’.

